# Response letter: “Predicted long-term antibody persistence for a tick-borne encephalitis vaccine: results from a modeling study beyond 10 years after a booster dose following different primary vaccination schedules”

**DOI:** 10.1080/21645515.2020.1798713

**Published:** 2020-08-18

**Authors:** Marco Costantini, Andrea Callegaro, Jiří Beran, Valérie Berlaimont, Ilaria Galgani

**Affiliations:** a GSK, Siena, Italy; b GSK, Rixensart, Belgium; c Vaccination and Travel Medicine Centre, Hradec Králové, Czechia; d Department for Tropical, Travel Medicine and Immunization, Institute for Postgraduate Medical Education, Prague, Czechia; e GSK, Wavre, Belgium

**Keywords:** tick-borne encephalitis, vaccination, antibody persistence, neutralizing titer, power-law models

Dear Dr Ellis,

We respond to a recent critique by Khan and colleagues, pertaining to the work of Costantini et al. titled “Predicted long-term antibody persistence for a tick-borne encephalitis vaccine: results from a modeling study beyond 10 years after a booster dose following different primary vaccine schedules.”^1^ In their letter, Khan and colleagues raise several concerns regarding the methodology used while proposing an alternative approach based on different assumptions. They provide a custom modeling approach on published data, to predict the decay of antibody titers in TBE vaccine recipients up to 20 y after the booster dose. Despite its constructive aspect, we believe this criticism is largely unfounded. We provide our responses to the individual points below.

1. One of the raised concerns was that the presented forecasts potentially overestimate antibody persistence to TBE vaccine, as the data were likely not censored properly. Khan and coauthors also state that the antibody decay estimations may be skewed, as only 48% of initial subjects were followed up for 10 y.

We would first like to clarify that there was no selection bias and no intent to enroll only the best responders, in terms of immunogenicity. As a matter of fact, subjects who did and those who did not participate in the first extension study, namely V48P7E1, showed similar geometric mean titers (GMTs) at Visit 8 (d 300, all groups combined as done in the modeling exercise), as clearly demonstrated by previously unpublished GMT values herein reported: The measured GMTs were 19.66 in subjects who discontinued participation after the initial study (V48P7) and 17.27 in subjects who continued in the V48P7E1. There is no reason to believe that the study dropout was somehow linked to the antibody level at the time of study withdrawal, as the missing data mechanism was completely at random (MCAR).

The probability of being lost to follow-up was identical for all subjects, irrespective of their antibody levels. Subjects were included in the extension studies only if they consented, they were healthy and had fulfilled the protocol and vaccination requirements in the previous study, without having reported important protocol deviations. Failure to fulfill one or more of these requirements led to subject exclusion. Additionally, 133 subjects from the original V48P7 study who were vaccinated with the modified conventional schedule were excluded from the study extensions, because this schedule was not marketed. Therefore, the 191 subjects described were actually representative of the eligible population. Importantly, the majority of subjects who were included in the extension studies regularly attended blood sampling visits until y 10 post-booster dose (and beyond, as a third extension is currently ongoing with a drop-out rate of below 10%).

Very few subjects (n = 4) were excluded from study extensions because of ethical standards as their NT levels were below 10 at the end of the parent study, and therefore, they were due to receive a further TBE vaccine dose. The impact of those four subjects on our modeling exercise is minimal, if any at all, and there should not be any concern on the potential bias for the prediction analysis.^2,3^ For further details on inclusion and exclusion criteria, please refer to the detailed study protocols at clinicaltrials.gov.

2. The following point of criticism was that the power-law models (PLMs) were incorrectly used, as they did not account for aging and its effect on antibody decay over time.

While the basic PLMs were indeed originally applied to a homogeneous population,^4^ we disagree with Khan and collaborators that these models are unsuitable for the population we selected in our latest study.

As discussed in a previous publication,^2^ it is true that relatively lower GMTs were observed in subjects ≥50 y of age compared to younger individuals. Tables 1 and 2 show antibody profiles by age from the V48P7 study. In addition, Table 3 clearly shows a distinction in GMT values between the following age groups: <50, ≥50 and ≥60 y. Note that, even though GMT values were on average lower for the latter group of subjects, they were still much higher than the protective level of 10 (GMTs ≥57 in all age groups until y 10) and the GMT decrease was not more pronounced with increased age. Still, we acknowledge that the described PLMs may be of limited applicability for individuals above 50 y of age, due to their low participation numbers (Table 3). Of the 213 subjects included in our modeling analysis, only 40 (18.8%) were aged 50 or older, of which 7 (3.3%) were 60 y of age or older.

**Table 1.** Percentage of subjects with TBE NT ≥10 at d 300 (visit 8) – PP set.

Age group (years)	TBE_R, N (%)	TBE_C, N (%)	TBE_AC, N (%)
12–17	13 (100)	10 (71)	19 (86)
18–49	27 (75)	25 (74)	36 (51)
≥50	7 (50)	7 (64)	10 (56)
≥60	1 (33)	1 (100)	2 (50)

TBE, tick-borne encephalitis; N (%), number (percentage) of subjects; NT, neutralizing titer; PP, per-protocol; TBE_R, TBE_C and TBE_AC, rapid, conventional and accelerated conventional TBE vaccination schedules in the V48P7 study.

**Table 2.** GMTs at d 300 (visit 8) – PP set.

	GMT (95% CI)		
Age group (years)	TBE_R	TBE_C	TBE_AC
12–17	47 (25–86)	35 (20–63)	33 (21–52)
18–49	18 (13–25)	21 (15–29)	12 (9–15)
≥50	13 (7–24)	13 (7–26)	11 (6–19)
≥60	14 (6–33)	32 (7–141)	9 (4–18)

GMT, geometric mean titer; TBE, tick-borne encephalitis; PP, per-protocol; TBE_R, TBE_C and TBE_AC, rapid, conventional and accelerated conventional TBE vaccination schedules in the V48P7 study.

**Table 3.** GMTs per visit and age group – PP set.

			TBE_R		TBE_C		TBE_AC
Age group (years)	Visit	N	GMT (95% CI)	N	GMT (95% CI)	N	GMT (95% CI)
15–49	P.b. V48P7E1	35	638 (413–984)	39	1299 (861–1959)	80	1008 (757–1343)
	Visit 18 (Y 6)	32	456 (278–747)	39	341 (218–533)	77	295 (215–406)
	Visit 19 (Y 7)	31	477 (300–760)	39	384 (254–582)	76	359 (267–483)
	Visit 20 (Y 8)	31	289 (180–464)	38	252 (164–386)	75	239 (177–325)
	Visit 21 (Y 9)	31	436 (270–704)	36	330 (211–514)	74	314 (231–429)
	Visit 22 (Y 10)	31	369 (217–628)	37	359 (221–585)	75	306 (217–430)
≥50	P.b. V48P7E1	13	201 (111–364)	12	733 (395–1361)	25	914 (595–1403)
	Visit 18 (Y 6)	13	207 (107–401)	12	178 (89–355)	25	183 (114–295)
	Visit 19 (Y 7)	12	244 (109–548)	12	237 (106–533)	24	267 (151–473)
	Visit 20 (Y 8)	12	143 (66–309)	12	189 (88–409)	24	174 (101–300)
	Visit 21 (Y 9)	12	299 (137–650)	12	224 (103–486)	24	251 (145–436)
	Visit 22 (Y 10)	12	178 (82–388)	12	189 (87–411)	24	157 (91–273)
≥60	P.b. V48P7E1	4	173 (71–425)	3	573 (203–1616)	7	498 (253–982)
	Visit 18 (Y 6)	4	189 (52–688)	3	96 (22–427)	7	76 (29–202)
	Visit 19 (Y 7)	4	133 (26–677)	3	120 (18–789)	7	105 (31–360)
	Visit 20 (Y 8)	4	128 (26–620)	3	81 (13–504)	7	71 (21–234)
	Visit 21 (Y 9)	4	224 (52–965)	3	67 (12–362)	7	128 (42–385)
	Visit 22 (Y 10)	4	181 (35–928)	3	57 (9–377)	7	72 (21–248)

GMT, geometric mean titer; TBE, tick-borne encephalitis; PP, per-protocol; TBE_R, TBE_C and TBE_AC, rapid, conventional and accelerated conventional TBE vaccination schedules in the V48P7 study; P.b., baseline post-booster.

Further data will be instructive in deciding to which extent these models may be used in the population of ≥50-y olds. However, we consider that the modeling data is applicable to all other analyzed age groups. Even though the sample size was small, these results implied that long-term immunity persists up to 10 y after vaccination in individuals older than 50 y. This GMT persistence may in turn point toward an extension of the booster intervals for elderly individuals. Furthermore, based on the clinical data obtained after 12 y of continuous follow-up (data not published yet), it appears that protection against TBE is long-lasting, regardless of the applied vaccination schedule and age at immunization, pointing toward a confirmation of the current modeling exercise.

3. In their final point of criticism, Khan and colleagues are referring to a publication by Beck et al. which looked at cross-protection against heterologous European TBE strains with TBE vaccine.

Although we acknowledge the existence of the data published by Beck and colleagues in 2015,^5^ which have been generated ex-vivo using a hybrid virus assay platform, reference to any specific publication from the German National Reference Center seems to be missing in this response. It is therefore not possible to confirm the statement that adequate protection against TBE virus (TBEV) strains may not be provided by Encepur, nor that this would be resulting from a mutation in the K23 seed virus.

The study by Beck et al.^5^ described a difference in neutralizing antibody titers against both virus strains (homologous K23 and heterologous Neudoerfl) observed between the sera from individuals vaccinated with Encepur-Children or FSME-Immun Junior. However, despite this observed difference, we would like to point out that the seropositivity rates against the heterologous Neudoerfl strain reached ≥94% after vaccination with Encepur-Children, ensuring protection to a large majority of the subjects. To our knowledge, this study did not test any other heterologous strain for any of the two vaccines and does not allow to conclude on the protection they offer against other TBEV strains.

A similarly high protection rate against heterologous TBE strains has been observed after vaccination with Encepur-Children in other studies. In particular, in a clinical study comparing the two European vaccines, a significantly higher percentage of children achieved neutralizing antibody titers ≥10 (an accepted surrogate marker of protection) after two doses of Encepur-Children against both the K23 and the Neudoerfl strains in neutralization tests using the respective strains, compared to FSME-Immun Junior.^6^ A third dose of Encepur-Children given to the FSME group increased the protection rate to close to 100%.

Other comparative studies found no difference between recipients of both vaccines in an NT-based assay on Neudoerfl strain with regard to detectable antibody titers after the second dose.^7,8^ A third dose of FSME-Immun Junior increased the protective rate to 100% in both groups.

In addition, a study performed soon after the development of the TBE vaccine demonstrated that sera of human vaccinees with Encepur were capable of neutralizing the 11 heterologous TBEV isolates tested.^9^ A more recent systematic literature review by Domnich and colleagues in 2014 suggests that both Western vaccines induce similar seropositivity rates against various strains of heterologous subtypes and that an immune response is developed against various TBEV strains of Far-Eastern and Siberian origin after vaccination with Encepur (Adult and Children).^10^

Should a significant difference in protection have been confirmed between the two vaccines, which have been on the market for more than 15 y, this would certainly have been reported as an imbalance of breakthrough cases in fully vaccinated individuals. However, those reports remain rare.^11^

Based on the data described above, the World Health Organization (WHO) considers both vaccines safe and efficacious for individuals ≥1 y of age. According to WHO, both vaccines appear to protect against all virus subtypes circulating in endemic areas in Europe and Asia, and they can be used interchangeably.^12^
